# Pulmonary Surfactant Treatment for Preterm Infants With Respiratory Distress Syndrome: A Nationwide Survey

**DOI:** 10.1111/crj.70228

**Published:** 2026-09-01

**Authors:** Haifeng Zong, Chuanzhong Yang, Long Chen, Yong Ji, Rong Ju, Jiangqin Liu, Ling Liu, Jingyun Shi, Hui Wu, Lili Wang, Falin Xu, Huayan Zhang, Yuan Shi, MUNICH Study Group

**Affiliations:** ^1^ Department of Neonatology Shenzhen Maternity & Child Healthcare Hospital Shenzhen Guangdong China; ^2^ Department of Neonatology Women and Children's Hospital of Chongqing Medical University, Chongqing Health Center for Women and Children Chongqing China; ^3^ Department of Neonatology Children's Hospital of Shanxi Taiyuan Shanxi China; ^4^ Department of Neonatology Chengdu Women's and Children's Central Hospital Chengdu Sichuan China; ^5^ Department of Neonatology Shanghai First Maternity and Infant Hospital, Tongji University School of Medicine Shanghai China; ^6^ Department of Neonatology Guiyang Maternal and Child Health Care Hospital‐Guiyang Children's Hospital Guiyang Guizhou China; ^7^ Department of Neonatology Gansu Provincial Maternal and Child Care Hospital (Gansu Provincial Central Hospital) Lanzhou Gansu China; ^8^ Department of Neonatology The First Hospital of Jilin University Changchun Jilin China; ^9^ Department of Neonatology The First Affiliated Hospital of Anhui Medical University Hefei Anhui China; ^10^ Department of Neonatology The Third Affiliated Hospital of Zhengzhou University Zhengzhou Henan China; ^11^ Department of Neonatology Guangzhou Women and Children's Medical Center, National Children's Medical Center for South Central Region Guangzhou Guangdong China; ^12^ Division of Neonatology Children's Hospital of Philadelphia Philadelphia Pennsylvania USA; ^13^ Department of Neonatology Children's Hospital of Chongqing Medical University, National Clinical Research Center for Child Health and Disorders, Ministry of Education Key Laboratory of Child Development and Disorders Chongqing China

**Keywords:** calsurf, neonatal respiratory distress syndrome, poractant alfa, pulmonary surfactant, survey

## Abstract

**Background:**

Pulmonary surfactant is essential for neonatal respiratory distress syndrome (RDS) management, yet national data on how surfactant is used in China are limited. We surveyed current practices and variation in surfactant availability and administration.

**Methods:**

In October–December 2022, a nationwide cross‐sectional online survey was completed by one neonatologist from each of 394 hospitals across 30 provinces. Items covered surfactant availability (including delivery room access), product selection, dosing, timing, and administration techniques.

**Results:**

Surfactant was available in 96.7% of hospitals; poractant alfa and calsurf were available in 88.6% and 58.1%, respectively. Only 51.6% of hospitals reported surfactant availability in delivery rooms. For the first dose, 200 mg/kg was preferred for poractant alfa (68.8%) and 100 mg/kg for calsurf (63.8%). The reported median time to first dose was 2.0 h after birth; poractant alfa was administered sooner than calsurf (10 vs. 30 min estimated administration time; *p* < 0.001). Most respondents (79.7%) reported giving surfactant after noninvasive ventilation failure in infants who subsequently required mechanical ventilation, with higher use in tertiary vs. secondary hospitals (82.1% vs. 71.7%; *p* = 0.03). Intubation‐surfactant‐extubation (INSURE) and less invasive surfactant administration (LISA)/minimally invasive surfactant therapy (MIST) were available in 86.5% and 49.7% of hospitals, respectively. Additionally, 25.1% of the respondents reported combining a surfactant with glucocorticoids for treating RDS.

**Conclusion:**

Surfactant use in China shows substantial between‐hospital variation, particularly in delivery room access and less invasive administration. Standardized training and improved resource allocation may reduce disparities and support timely, evidence‐based surfactant therapy.

## Graphical abstract

A nationwide survey across 394 hospitals in China revealed substantial variation in pulmonary surfactant administration for neonatal respiratory distress syndrome. Delivery room access to surfactant and adoption of less invasive techniques such as LISA/MIST remained limited, particularly in lower‐level hospitals, highlighting the need for standardized respiratory management and training strategies.

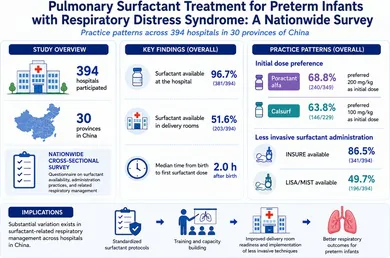

## 1 Introduction

Neonatal respiratory distress syndrome (RDS), a prevalent lung disorder in newborns, arises from a deficiency in pulmonary surfactant and/or immature lung development [1, 2, 3]. Surfactant therapy in RDS patients reduces mortality and pulmonary air leakage and plays an important role in the management of RDS [4, 5, 6].

Because the first launch of surfactant therapy in Europe in 1992, its timing and method of administration have changed significantly over the past three decades [7]. Clinical recommendations have largely shifted away from the use of a prophylactic surfactant toward surfactant use in only infants who exhibit clinical signs of RDS. Concurrently, methods of administration have advanced; less invasive surfactant administration (LISA), which uses thin catheters proposed for the treatment of spontaneously breathing infants on continuous positive airway pressure (CPAP), has emerged as an alternative to standard surfactant replacement therapy (SRT) [8].

Guidelines and consensus statements on RDS management, such as those from the European Consensus and Canadian guidelines, focus on determining when and how to use pulmonary surfactants as well as the optimal surfactant [9, 10, 11, 12, 13]. However, although the main elements of these guidelines are broadly consistent, small differences in the recommendations can lead to clinical variability. For example, the European guidelines recommend treating worsening RDS with surfactant when the fraction of inspired oxygen (FiO_2_) is > 0.3 at a CPAP pressure ≥ 6 cm H_2_O, whereas the Canadian guidelines recommend a higher threshold of FiO_2_ > 0.5 [13]. The clinical practice of surfactant treatment may differ as a result of these differences.

In China, surfactant use has spanned more than two decades, during which poractant alfa first introduced in 2001, followed by calsurf (a local bovine surfactant) in 2005. Despite the common use of these two products, detailed national data concerning the specific practices—when, which, and how—of their administration across the country are lacking. To address this gap, a nationwide, multicenter, cross‐sectional survey, the medical survey of natal intensive care unit (NICU) insight in China (MUNICH), was conducted.

## 2 Material and Methods

### 2.1 Study Design and Participants

This was a nationwide cross‐sectional survey conducted as part of the Medical Survey of NICU Insight in China (MUNICH) project [14]. The survey was performed between October and December 2022 and included one neonatologist from each participating hospital across 30 provinces in China.

Hospitals were invited through professional neonatal networks. Each hospital designated one representative physician to complete the questionnaire to reflect institutional practice patterns. Participation was voluntary and anonymous.

This study was conducted in accordance with the Declaration of Helsinki and was approved by the Ethics Committee of the Children's Hospital of Chongqing Medical University (Approval Number: 2022–376). All participating physicians provided informed consent prior to survey completion. Race and ethnicity data were not collected in this survey because the study focused on institutional clinical practice patterns rather than patient‐level characteristics.

### 2.2 Survey Instrument

**FIGURE 1:**
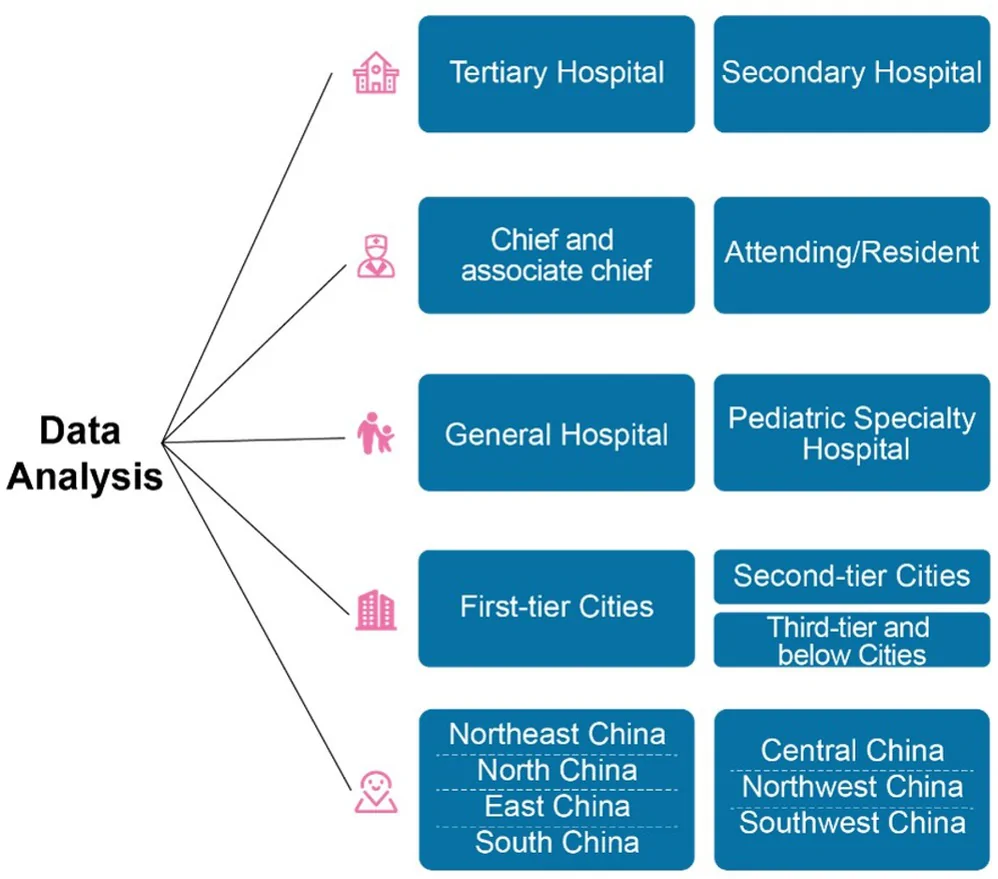
Data analysis dimensions.

The questionnaire comprised more than 80 items covering six domains: general information, prenatal and perinatal conditions, noninvasive ventilation, mechanical ventilation, pulmonary surfactant administration, and related clinical practices (Supporting Information). Questions specifically addressed surfactant availability, delivery room access, initial dosing practices, timing of first and second dose, indications for surfactant use, and administration techniques (intubation‐surfactant‐extubation [INSURE], LISA/minimally invasive surfactant therapy [MIST]). Hospitals were stratified by level (tertiary vs. secondary), city tier, and geographic region (Figure 1).

### 2.3 Statistical Analysis

Categorical variables are presented as frequencies and percentages. Continuous variables are reported as medians with interquartile ranges (IQR). Comparisons between groups were performed using chi‐square tests for categorical variables and nonparametric tests for continuous variables as appropriate. A two‐sided *p*‐value < 0.05 was considered statistically significant.

## 3 Results

### 3.1 Characteristics of the Respondents

A total of 394 valid responses (one physician from each hospital) were obtained from neonatologists across 30 provinces in China. The demographic characteristics of the participants and hospitals are detailed in the supplemental materials, as shown in Table S1.

### 3.2 Surfactant Accessibility in Hospitals

Surfactants were available in 96.7% (381/394) of the hospitals surveyed (poractant alfa was available in 88.6% of the hospitals, and calsurf was available in 58.1% of the hospitals). Among these hospitals, 197 hospitals had both surfactants, 152 had poractant alfa only, and 32 had calsurf only. The availability of both products was significantly greater in tertiary hospitals than in secondary hospitals (poractant alfa, 91.1% vs. 80.4%, *p* = 0.005; calsurf, 65.6% vs. 33.7%, *p* < 0.001). Poractant alfa was also more readily available in pediatric specialty hospitals (93.5% vs. 85.5%, *p* = 0.015) and in higher tier cities (*p* = 0.013) (Table 1). The distribution of calsurf differed across regions (*p* = 0.010) (Table S2).

**TABLE 1:** Availability of surfactants in different hospitals.

	*n* = 394	Hospital tier			Hospital type			City tier			
		Tertiary hospital	Secondary hospital	*p*	General hospital	Pediatric specialty hospital	*p*	1st‐tier cities	2nd‐tier cities	3rd‐tier and lower tier cities	*p*
**Poractant alfa,** *n* (%)	349 (88.6)	275 (91.1)	74 (80.4)	0.005	206 (85.5)	143 (93.5)	0.015	123 (93.9)	51 (92.7)	175 (84.1)	0.013
**Calsurf,** *n* (%)	229 (58.1)	198 (65.6)	31 (33.7)	<0.001	138 (57.3)	91 (59.5)	0.664	73 (55.7)	33 (60.0)	123 (59.1)	0.788
**No surfactant,** *n* (%)	13 (3.3)	3 (1.0)	10 (10.9)	<0.001	9 (3.7)	4 (2.6)	0.544	1 (0.8)	0	12 (5.8)	0.014

### 3.3 Surfactant in Delivery Rooms (DRs)

**FIGURE 2:**
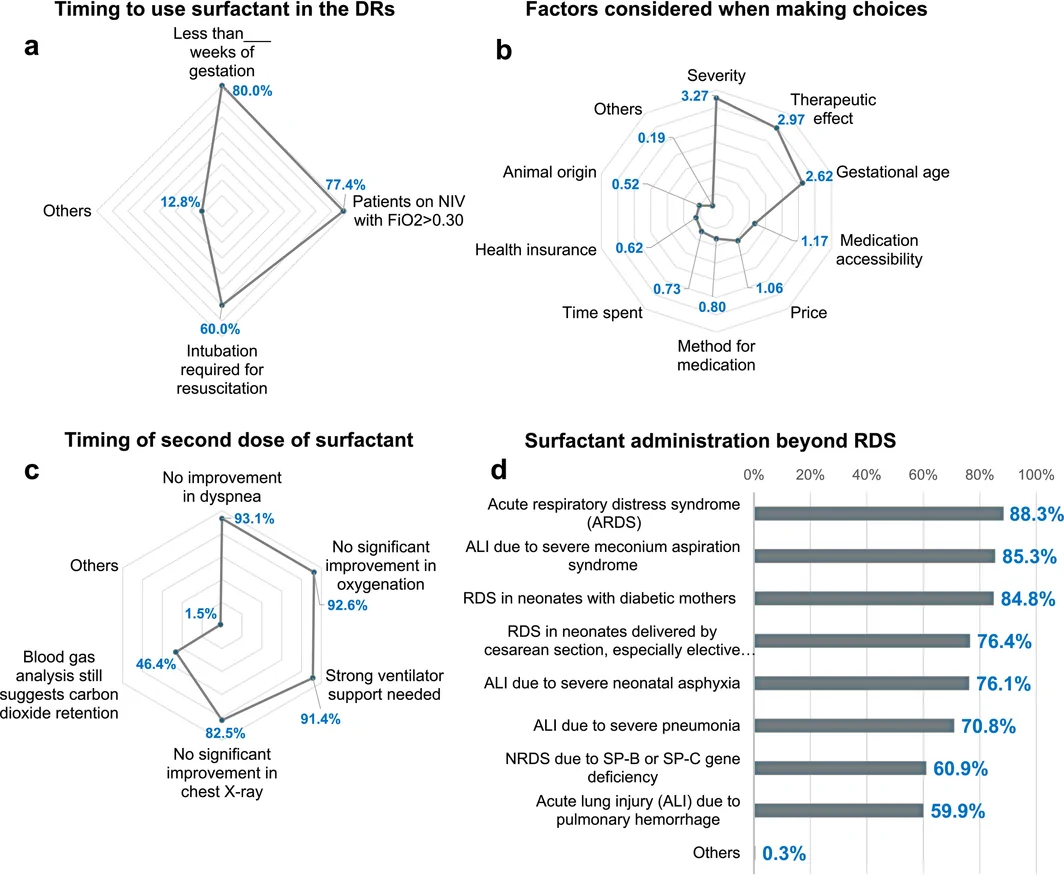
(a) Timing of surfactant use in the DRs. (b) Factors considered when making choices. (c) Timing of the second dose of surfactant. (d) Surfactant administration beyond RDS. Abbreviations: DRs, delivery rooms; RDS, respiratory distress syndrome.

Surfactants were available in the DRs of only 195 (51.6%) hospitals. Surfactants were administered in the DR on the basis of key criteria including gestational age (80.0%), FiO2 > 0.30 for patients on noninvasive ventilation (77.4%), and the need for intubation for resuscitation (60.0%), as shown in Figure 2a. The median threshold for consideration of surfactant use among the survey doctors was a gestational age of less than 28.0 (28.0, 29.0) weeks.

### 3.4 Administration of Surfactant

#### 3.4.1 Dose

For poractant alfa, 68.8% (240/349) of the respondents chose 200 mg/kg as the initial dose. This dosing approach differed significantly between tertiary and secondary hospitals (*p* = 0.002). With respect to calsurf, 63.8% (146/229) of the doctors reported administering 100 mg/kg as the first dose. This dosing approach differed between general and pediatric specialty hospitals (*p* = 0.002) (Table 2).

**TABLE 2:** Initial dose of surfactants in different hospitals.

		Hospital tier			Hospital type			City tier			
		Tertiary hospital	Secondary hospital	*p*	General hospital	Pediatric specialty hospital	*p*	1st‐tier cities	2nd‐tier cities	3rd‐tier and lower tier cities	*p*
**Poractant alfa**	***N* = 349**										
200 mg/kg	240 (68.8)	201 (73.1)	39 (52.7)	0.002	139 (67.5)	101 (70.6)	0.798	78 (63.4)	35 (68.6)	127 (72.6)	0.495
100‐200 mg/kg	103 (29.5)	70 (25.5)	33 (44.6)		63 (30.6)	40 (28.0)		42 (34.1)	16 (31.4)	45 (25.7)	
100 mg/kg	5 (1.4)	4 (1.5)	1 (1.4)		3 (1.5)	2 (1.4)		3 (2.4)	0	2 (1.1)	
<100 mg/kg	1 (0.3)	0	1 (1.4)		1 (0.5)	0		0	0	1 (0.6)	
>200 mg/kg	0	0	0		0	0		0	0	0	
**Calsurf**	***N* = 229**										
100 mg/kg	146 (63.8)	130 (65.7)	16 (51.6)	0.270	89 (64.5)	57 (62.6)	0.004	46 (63.0)	18 (54.5)	82 (66.7)	0.414
70 mg/kg	62 (27.1)	51 (25.8)	11 (35.5)		30 (21.7)	32 (35.2)		21 (28.8)	9 (27.3)	32 (26.0)	
40 mg/kg	0	0	0		0	0		0	0	0	
40‐100 mg/kg	14 (6.1)	12 (6.1)	2 (6.5)		14 (10.1)	0		4 (5.5)	5 (15.2)	5 (4.1)	
>100 mg/kg	7 (3.1)	5 (2.5)	2 (6.5)		5 (3.6)	2 (2.2)		2 (2.7)	1 (3.0)	4 (3.3)	
<40 mg/kg	0	0	0		0	0		0	0	0	

#### 3.4.2 Considerations

When choosing between the two available products, physicians reported that the main considerations were disease severity, therapeutic effect, gestational age (GA), accessibility, and price (Figure 2b). Among the 197 respondents with access to both surfactants, poractant alfa was preferred for preterm infants with a median GA of less than 30.0 (28.0, 32.0) weeks.

#### 3.4.3 Methods

When treating patients indicated for surfactant use while on noninvasive ventilation (NIV), 86.5% (341/394) of the respondents could perform the INSURE method. However, only 49.7% (196/394) of the respondents could administer the LISA and/or MIST (Table 3).

**TABLE 3:** Methods of surfactant administration to patients on noninvasive ventilation.

	*n* = 394	Hospital tier			Hospital type			City tier			
		Tertiary hospital	Secondary hospital	*p*	General hospital	Pediatric specialty hospital	*p*	1st‐tier cities	2nd‐tier cities	3rd‐tier and lower tier cities	*p*
**INSURE**	341 (86.5)	262 (86.8)	79 (85.9)	0.828	213 (88.4)	128 (83.7)	0.181	110 (84.0)	47 (85.5)	184 (88.5)	0.482
**LISA/MIST**	196 (49.7)	167 (55.3)	29 (31.5)	<0.001	108 (44.8)	88 (57.5)	0.014	69 (52.7)	30 (54.5)	97 (46.6)	0.415
**Use surfactant and switch to MV**	202 (51.3)	165 (54.6)	37 (40.2)	0.015	124 (51.5)	78 (51.0)	0.927	71 (54.2)	25 (45.5)	106 (51.0)	0.548
**Others**	11 (2.8)	5 (1.7)	6 (6.5)	0.013	7 (2.9)	4 (2.6)	0.865	0	0	11 (5.3)	0.006

Abbreviations: INSURE, intubation‐surfactant‐extubation; LISA/MIST, less invasive surfactant administration/minimally invasive surfactant therapy; MV, mechanical ventilation.

#### 3.4.4 Timing of First Dose

Physicians reported that the first dose should be given at a median time of 2.0 h after birth. A critical disparity in timing was observed for poractant alfa administration: Physicians in secondary hospitals tended to use poractant alfa significantly later (5.5 (2.0, 6.0) h) than those in tertiary hospitals did (2.0 (1.0, 6.0) h, *p* = 0.020) (Table 4). Surfactants were routinely administered after NIV failure in patients requiring endotracheal intubation and mechanical ventilation (MV), as reported by 79.7% of the respondents (82.1% of those in tertiary hospitals vs. 71.7% of those in secondary hospitals, *p* = 0.03). A total of 60.4% (238/394) of the respondents used FiO_2_ as an indicator for surfactant administration for patients receiving NIV, with no differences found between hospitals. Among these respondents, 41.6% chose FiO_2_ > 0.30 as the threshold for surfactant administration (Table S3). When performing LISA/MIST, 50.5% (199/394) of the respondents chose a feeding tube, 28.2% chose a peripheral venous catheter, and 19.8% chose an umbilical venous catheter as the administration route.

**TABLE 4:** Administration of poractant alfa and calsurf in different hospitals.

		Hospital tier			Hospital type			City tier			
		Tertiary hospital	Secondary hospital	*p*	General hospital	Pediatric specialty hospital	*p*	1st‐tier cities	2nd‐tier cities	3rd‐tier and lower tier cities	*p*
**Poractant alfa**	***N* = 349**	***N* = 275**	***N* = 74**		***N* = 206**	***N* = 143**		***N* = 123**	***N* = 51**	***N* = 175**	
1st dose, hours Median (Q1, Q3)	2.0 (2.0, 6.0)	2.0 (1.0, 6.0)	5.5 (2.0, 6.0)	0.020	2.0 (2.0, 6.0)	2.0 (1.5, 6.0)	0.894	2.0 (2.0, 6.0)	2.0 (1.0, 6.0)	2.0 (2.0, 6.0)	0.862
Injection time, min Median (Q1, Q3)	10.0 (5.0, 15.0)	10.0 (5.0, 20.0)	10.0 (5.0, 15.0)	0.755	10.0 (5.0, 20.0)	10.0 (5.0, 15.0)	0.729	10.0 (5.0, 20.0)	15.0 (5.0, 20.0)	10.0 (5.0, 15.0)	0.096
**Calsurf**	***N* = 229**	***N* = 198**	***N* = 31**		***N* = 138**	***N* = 91**		***N* = 73**	***N* = 33**	***N* = 123**	
1st dose, hours Median (Q1, Q3)	2.0 (1.0, 6.0)	2.0 (2.0, 6.0)	2.0 (1.0, 6.0)	0.488	2.0 (2.0, 6.0)	2.0 (1.0, 6.0)	0.932	2.0 (1.0, 6.0)	2.0 (1.0, 6.0)	2.0 (2.0, 6.0)	0.969
Injectio*n* time, min Median (Q1, Q3)	30.0 (15.0, 30.0)	30.0 (15.0, 33.75)	20.0 (15.0, 30.0)	0.149	30.0 (13.12, 30.0)	30.0 (15.0, 32.5)	0.345	20.0 (10.0, 30.0)	30.0 (30.0, 40.0)	30.0 (15.0, 30.0)	0.034

Abbreviations: Q1, first quartile; Q3, third quartile.

#### 3.4.5 Timing of Second Dose

Indicators for a second dose included a lack of improvement in dyspnea (93.1%), inadequate improvement in oxygenation (92.6%), a persistent need for ventilator support (91.4%), and no evident improvement on chest X‐ray (82.5%) (Figure 2c). The median time interval considered for a second dose was 9.0 (6.0, 12.0) h. Compared with those in tertiary hospitals, doctors in secondary hospitals tended to report a longer time interval for redosing (12.0 vs. 9.0 h).

#### 3.4.6 Injection Time

The estimated administration time for poractant alfa was significantly shorter (10.0 (5.0, 15.0) min) than that for calsurf (30.0 (15.0, 30.0) min) (*p* < 0.001). Furthermore, doctors in 2nd‐tier cities and 3rd‐tier and lower cities reported a longer duration of calsurf administration (Table 4).

### 3.5 Surfactant and Glucocorticoid

A combination of a surfactant and glucocorticoids was used by 99 (25.1%) of the respondents. Among these, 65.7% chose to use glucocorticoids with the first dose of surfactant.

### 3.6 Surfactant Therapy Beyond RDS

Surfactants were also commonly used in conditions other than primary RDS. The most common uses were for acute respiratory distress syndrome (ARDS) (88.3%), acute lung injury (ALI) caused by severe meconium aspiration syndrome (85.3%), and neonatal respiratory distress syndrome (NRDS) in neonates of diabetic mothers (84.8%) (Figure 2d).

## 4 Discussion

SRT has become routine and essential for infants diagnosed with RDS. This national survey aimed to comprehensively investigate the administration of surfactant across China and analyze the significant heterogeneity associated with its use. The results reveal a wide variety in surfactant use across NICU centers, reflecting substantial variability in neonatologists' attitudes and practices, which can be attributed primarily to a lack of homogeneous training and unbalanced allocation of resources among different hospitals.

Surfactants were available in almost every hospital (96.7%), whereas accessibility remained suboptimal in critical areas. Specifically, surfactant was available in the DRs of only 51.6% of the hospitals. The availability of specific products also reflected geographical and financial disparities: Poractant alfa (88.6% availability) was more readily accessible in higher level cities, whereas calsurf (58.1% availability), which is reported to have a lower cost, was more common in lower tier cities. When choosing between these two products, physicians considered disease severity, therapeutic effect, GA, accessibility, and price. Poractant alfa was preferred for preterm infants with a median GA of less than 30.0 weeks.

Nearly two‐thirds (68.8%) of the respondents chose 200 mg/kg for the initial dose of poractant alfa. This choice aligns with the European Consensus Guidelines' recommendation for 200 mg/kg of poractant alfa as the preferred initial dose for rescue therapy [9]. Nevertheless, variability in surfactant dosing has also been reported in European clinical practice. Jourdain et al. reported deviations from the recommended surfactant dose among preterm infants with RDS [15], and a multicenter study from Spain found that only 47.5% of preterm infants treated with poractant alfa received an initial dose within 10% of the recommended 200 mg/kg, whereas 47.0% received less than 180 mg/kg [16]. These findings indicate that adherence to recommended surfactant dosing remains a challenge across different healthcare settings. Cornette et al. similarly highlighted that although a correct surfactant dose of 200 mg/kg is recommended, it is not always adhered to, leading to risks associated with both under‐ and overtreatment of infants. Undertreating small infants for financial reasons must be avoided, as it increases the likelihood of the need for a second dose [17]. Inappropriate surfactant dosing may therefore have implications beyond treatment efficacy and has also been discussed as a medicolegal and cultural issue in neonatal care [18]. Ensuring adherence to evidence‐based dosing recommendations is important, as surfactant dose and pharmacokinetics may influence the need for subsequent redosing [19]. With respect to calsurf, 63.8% of the doctors administered an initial dose of 100 mg/kg, which differed between physicians at general and pediatric specialty hospitals. A total of 35.2% of the doctors in pediatric specialty hospitals chose 70 mg/kg as the initial dose of calsurf.

There are three main modes for administering surfactant in patients on NIV: INSURE, LISA/MIST, and MV after surfactant infusion. Studies have demonstrated that the LISA technique offers significant benefits in the treatment of RDS, leading to a reduced CPAP duration, a lower need for MV, and a decreased incidence of bronchopulmonary dysplasia (BPD), pneumothorax, peri‐intraventricular hemorrhage and mortality compared to INSURE [20, 21]. On the basis of these compelling results, the LISA method has been shown to have potential clinical advantages for surfactant administration. In the current survey, INSURE was the preferred option, followed by LISA/MIST. The LISA/MIST method was used more frequently in tertiary hospitals than in secondary hospitals, which may be related to better professional training and access to clinical practice updates in high‐level medical centers. Similarly, compared with those in general hospitals, physicians in pediatric specialty hospitals reported a higher rate of LISA/MIST use. Notably, specialized catheters for performing LISA/MIST are lacking in China, which might partially explain the limited promotion of the two methods.

For both products, physicians universally believe that the first dose should be given at 2.0 h after birth, and 60.4% of the respondents used FiO_2_ as an indicator for surfactant administration in patients receiving NIV, with FiO_2_ > 0.30–0.4 as the threshold for surfactant treatment. Observational studies have consistently shown that infants requiring FiO_2_ > 0.3 within the first 2 h (while using CPAP up to 7–8 cm H_2_O) have a greater need for MV within 72 h [22, 23]. The European consensus guidelines also suggest a threshold of 0.3 FiO_2_ with a CPAP of at least 6 cm H_2_O [9]. Interestingly, compared with those in tertiary hospitals, doctors in secondary hospitals reported a significant delay in the use of poractant alfa. This discrepancy could be attributed to two potential factors. First, the patient population in secondary hospitals may comprise infants with an older GA and less severe clinical conditions; second, it might reflect a lack of relevant training. More than one dose of surfactant may be needed when there is no improvement in the clinical signs of respiratory distress, including persistent dyspnea, inadequate oxygenation, an ongoing need for significant ventilator support, and a lack of radiological improvement on chest X‐ray. The median time interval for the repeated surfactant dose was 9.0 h, which is consistent with the Chinese recommendation of 6 to 12 h after the initial dose [11]. Although there are no definitive guidelines for the injection duration, this survey reported median administration times of 10.0 min for poractant alfa and 30.0 min for calsurf, with significant regional variations observed.

In the current survey, 25.1% of the respondents reported using a combination of surfactant and glucocorticoid. Among these respondents, 65.7% administered glucocorticoid concomitantly with the first dose of surfactant. Previous trials have demonstrated that compared with surfactant alone, intratracheal glucocorticoids mixed with a surfactant are associated with a reduction in the risk of death or BPD of more than 33% [24, 25, 26]. In one of these trials, the prevalence of neurodevelopmental impairment at 2–3 years of age also potentially decreased [24]. On the basis of these findings, certain medical centers have incorporated intratracheal glucocorticoid into routine care [27]. Recently, an international randomized clinical trial assessing whether early intratracheal administration of a combination of glucocorticoid mixed with surfactant reduces physiologic BPD or death by 36 weeks postmenstrual age in extremely preterm infants revealed no reduction in the risk of BPD or death [28].

In addition to NRDS, surfactant therapy is also employed in other critical conditions characterized by secondary surfactant inactivation, including pneumonia [29], pulmonary hemorrhage [30] and meconium aspiration syndrome [31]. Consistent with this broader application, our survey indicated that surfactant was frequently used in patients with ARDS (88.3%), severe meconium aspiration syndrome (85.3%) and NRDS in neonates of diabetic mothers (84.8%).

This study has several limitations. First, as a questionnaire‐based survey, it may be subject to variances in clinical practice among physicians, even within the same hospital setting. Additionally, the questionnaire was completed by a single physician representing each unit, which may not fully capture the consensus or diversity of practices among all clinicians in the unit. Furthermore, issues such as incomplete medical insurance coverage and regional economic disparities could influence the generalizability of the results. Despite these limitations, the findings provide valuable insights that can inform the development of related public health policies and interventions.

## 5 Conclusion

This national survey revealed wide disparities in the use of surfactant. Poractant alfa is more commonly used, but generally, the use of surfactant varies considerably across different types of hospitals in China. The accessibility of surfactant in DRs and the routine administration of surfactant in MV patients should be improved. It is necessary to design and implement a formalized and accredited training program to reduce the current heterogeneity among centers.

## Author Contributions

**Haifeng Zong:** writing – original draft, methodology, data curation, formal analysis. **Chuanzhong Yang:** writing – review and editing, conceptualization/design, methodology, supervision/oversight. **Long Chen:** writing and review and editing. **Yong Ji:** writing – review and editing, writing – original draft, investigation, formal analysis. **Rong Ju:** writing – review and editing, methodology, data curation, formal analysis. **Jiangqin Liu:** writing – review and editing, methodology, data curation, formal analysis. **Ling Liu:** writing – review and editing, methodology, investigation, formal analysis. **Jingyun Shi:** writing – review and editing, original draft, investigation, formal analysis. **Hui Wu:** writing –review and editing, methodology, data curation, supervision/oversight. **Lili Wang:** writing–review and editing, writing – original draft, data curation, investigation. **Falin Xu:** writing – review and editing, writing – original draft, data curation, formal analysis. **Huayan Zhang:** writing – review and editing, conceptualization/design, methodology, supervision/oversight. **Yuan Shi:** writing – review and editing, conceptualization/design, methodology, supervision/oversight. All the authors reviewed the results and approved the final version of the manuscript. All the authors contributed equally.

## Funding

This survey was supported by the Chiesi China. Chiesi China played no role in the study design; the collection, analysis, or interpretation of the data; the writing of the report; or the decision to submit the paper for publication.

## Ethics Statement

This study was approved by the Ethics Committee of the Children's Hospital of Chongqing Medical University (Approval Number: 2022–376). All the physicians provided informed consent before they participated in the survey.

## Conflicts of Interest

All authors completed the ICMJE disclosure form. The study was funded by Chiesi China, which had no role in study design, data acquisition, analysis, interpretation, or publication decisions. All authors declare no additional conflicts of interest.

## Supporting information


**Table S1:** Characteristics of physicians and hospitals included in the survey.
**Table S2:** Availability of surfactants in different regions.
**Table S3:** FiO_2_ as a criterion for surfactant administration in patients under noninvasive ventilation in different hospitals.
**Data S1:** MUNICH questionnaire.

## Data Availability

The data that support the findings of this study are available from the corresponding author upon reasonable request. The data are not publicly available due to institutional and ethical restrictions.

## References

[1] D. G. Sweet , V. Carnielli , G. Greisen , et al., “European Consensus Guidelines on the Management of Respiratory Distress Syndrome—2019 Update,” Neonatology 115, no. 4 (2019): 432–450. 30974433 10.1159/000499361 PMC6604659

[2] C. L. Hermansen and A. Mahajan , “Newborn Respiratory Distress,” American Family Physician 92, no. 11 (2015): 994–1002. 26760414

[3] S. Yadav and B. Lee , “Neonatal Respiratory Distress Syndrome,” in StatPearls (StatPearls Publishing, 2023) [cited 2024 Mar 21]. http://www.ncbi.nlm.nih.gov/books/NBK560779/. PMID: 32809614. 32809614

[4] R. F. Soll , “Prophylactic Natural Surfactant Extract for Preventing Morbidity and Mortality in Preterm Infants,” Cochrane Database of Systematic Reviews 2 (2000): CD000511. 10.1002/14651858.CD000511 PMC7038705 10796380

[5] R. F. Soll , “Synthetic Surfactant for Respiratory Distress Syndrome in Preterm Infants,” Cochrane Database of Systematic Reviews 2 (2000): CD001149. 10.1002/14651858.CD001149 PMC8407430 10796417

[6] S. Sardesai , M. Biniwale , F. Wertheimer , A. Garingo , and R. Ramanathan , “Evolution of Surfactant Therapy for Respiratory Distress Syndrome: Past, Present, and Future,” Pediatric Research 81, no. 1–2 (2017): 240–248. 27706130 10.1038/pr.2016.203

[7] “First Approval for 'Curosurf',” Inpharma Weekly, (1992) 831, 23.

[8] M. E. Abdel‐Latif , P. G. Davis , K. I. Wheeler , A. G. De Paoli , and P. A. Dargaville , “Surfactant Therapy via Thin Catheter in Preterm Infants With or at Risk of Respiratory Distress Syndrome,” Cochrane Database of Systematic Reviews 5, no. 5 (2021): CD011672. 33970483 10.1002/14651858.CD011672.pub2 PMC8109227

[9] D. G. Sweet , V. P. Carnielli , G. Greisen , et al., “European Consensus Guidelines on the Management of Respiratory Distress Syndrome: 2022 Update,” Neonatology 120, no. 1 (2023): 3–23. 36863329 10.1159/000528914 PMC10064400

[10] R. A. Polin , W. A. Carlo , Committee on Fetus and Newborn , and American Academy of Pediatrics , “Surfactant Replacement Therapy for Preterm and Term Neonates With Respiratory Distress,” Pediatrics 133, no. 1 (2014): 156–163. 24379227 10.1542/peds.2013-3443

[11] Subspecialty Group of Neonatology, Society of Pediatrics, Chinese Medical Association and Editorial Board of Chinese Journal of Pediatrics , “Consensus for Pulmonary Surfactant Therapy in Neonates in China (2021),” Zhonghua Er Ke Za Zhi = Chinese Journal of Pediatrics 59, no. 8 (2021): 627–632. 34333913 10.3760/cma.j.cn112140-20210329-00261

[12] National Institute for Health and Care Excellence (NICE) , “Specialist Neonatal Respiratory Care for Babies Born Preterm. NICE Guideline NG124 [Internet],” (2019), https://www.nice.org.uk/guidance/ng124. 31194313

[13] E. H. Ng and V. Shah , “Guidelines for Surfactant Replacement Therapy in Neonates,” Paediatrics & Child Health 26, no. 1 (2021): 35–49. 33552321 10.1093/pch/pxaa116 PMC7850281

[14] L. Chen , Y. Ji , R. Ju , et al., “A Nationwide Survey on the Management of Neonatal Respiratory Distress Syndrome: Insights From the MUNICH Survey in 394 Chinese Hospitals,” Italian Journal of Pediatrics 50, no. 1 (2024): 168. 39244592 10.1186/s13052-024-01741-7 PMC11380405

[15] G. Jourdain , F. Zacaria , F. Ammar , and D. De Luca , “Appropriateness of Surfactant Dosing for Preterm Babies With Respiratory Distress Syndrome: Retrospective Cohort Study,” Archives of Disease in Childhood. Fetal and Neonatal Edition 101, no. 2 (2016): F182–F183. 26785859 10.1136/archdischild-2015-310195

[16] H. Boix , S. Rite , L. Arruza , et al., “Underdosing of Surfactant for Preterm Babies With Respiratory Distress Syndrome in Clinical Practice: A Retrospective Cohort Study,” American Journal of Perinatology 36, no. 09 (2019): 943–948. 30414597 10.1055/s-0038-1675645

[17] L. Cornette , A. Mulder , A. Debeer , et al., “Surfactant Use in Late Preterm Infants: A Survey Among Belgian Neonatologists,” European Journal of Pediatrics 180, no. 3 (2021): 885–892. 32970243 10.1007/s00431-020-03806-1 PMC7511270

[18] S. Foligno and D. De Luca , “Carelessness About Surfactant Dose—A Cultural Problem, a Legal Issue, or an Open Research Question?,” JAMA Pediatrics 173, no. 3 (2019): 211–212. 30615035 10.1001/jamapediatrics.2018.4296

[19] P. E. Cogo , M. Facco , M. Simonato , et al., “Pharmacokinetics and Clinical Predictors of Surfactant Redosing in Respiratory Distress Syndrome,” Intensive Care Medicine 37, no. 3 (2011): 510–517. 21153401 10.1007/s00134-010-2091-2

[20] R. C. Silveira , C. Panceri , N. P. Munoz , M. B. Carvalho , A. C. Fraga , and R. S. Procianoy , “Less Invasive Surfactant Administration Versus Intubation‐Surfactant‐Extubation in the Treatment of Neonatal Respiratory Distress Syndrome: A Systematic Review and Meta‐Analyses,” Jornal de Pediatria 100, no. 1 (2024): 8–24. 37353207 10.1016/j.jped.2023.05.008 PMC10751720

[21] I. Kuitunen and K. Räsänen , “Less Invasive Surfactant Administration Compared to Intubation, Surfactant, Rapid Extubation Method in Preterm Neonates: An Umbrella Review,” Neonatology 121, no. 4 (2024): 485–493. 38503270 10.1159/000537903 PMC11318579

[22] E. Gulczyńska , T. Szczapa , R. Hożejowski , M. K. Borszewska‐Kornacka , and M. Rutkowska , “Fraction of Inspired Oxygen as a Predictor of CPAP Failure in Preterm Infants With Respiratory Distress Syndrome: A Prospective Multicenter Study,” Neonatology 116, no. 2 (2019): 171–178. 31112987 10.1159/000499674 PMC6878744

[23] V. Kakkilaya , S. Wagner , K. L. M. Mangona , et al., “Early Predictors of Continuous Positive Airway Pressure Failure in Preterm Neonates,” Journal of Perinatology 39, no. 8 (2019): 1081–1088. 31089256 10.1038/s41372-019-0392-z

[24] T. F. Yeh , C. M. Chen , S. Y. Wu , et al., “Intratracheal Administration of Budesonide/Surfactant to Prevent Bronchopulmonary Dysplasia,” American Journal of Respiratory and Critical Care Medicine 193, no. 1 (2016): 86–95. 26351971 10.1164/rccm.201505-0861OC

[25] T. F. Yeh , H. C. Lin , C. H. Chang , et al., “Early Intratracheal Instillation of Budesonide Using Surfactant as a Vehicle to Prevent Chronic Lung Disease in Preterm Infants: A Pilot Study,” Pediatrics 121, no. 5 (2008): e1310–e1318. 18426851 10.1542/peds.2007-1973

[26] H. T. Kuo , H. C. Lin , C. H. Tsai , I. C. Chouc , and T. F. Yeh , “A Follow‐Up Study of Preterm Infants Given Budesonide Using Surfactant as a Vehicle to Prevent Chronic Lung Disease in Preterm Infants,” Journal of Pediatrics 156, no. 4 (2010): 537–541. 20138301 10.1016/j.jpeds.2009.10.049

[27] T. B. Kothe , F. H. Sadiq , N. Burleyson , H. L. Williams , C. Anderson , and N. H. Hillman , “Surfactant and Budesonide for Respiratory Distress Syndrome: An Observational Study,” Pediatric Research 87, no. 5 (2020): 940–945. 31715622 10.1038/s41390-019-0663-6

[28] N. Ambalavanan , W. A. Carlo , K. J. Nowak , et al., “Early Intratracheal Budesonide to Reduce Bronchopulmonary Dysplasia in Extremely Preterm Infants: The Budesonide in Babies (BiB) Randomized Clinical Trial,” JAMA 334 (2025): 1452–1462. 41026481 10.1001/jama.2025.16450 PMC12486137

[29] S. Deshpande , P. Suryawanshi , K. Ahya , R. Maheshwari , and S. Gupta , “Surfactant Therapy for Early Onset Pneumonia in Late Preterm and Term Neonates Needing Mechanical Ventilation,” Journal of Clinical and Diagnostic Research 11, no. 8 (2017): SC09–SC12. 28969229 10.7860/JCDR/2017/28523.10520 PMC5620870

[30] A. Aziz and A. Ohlsson , “Surfactant for Pulmonary Haemorrhage in Neonates,” Cochrane Database of Systematic Reviews 2, no. 2 (2020): CD005254. 32012227 10.1002/14651858.CD005254.pub4 PMC6996938

[31] A. I. El Shahed , P. A. Dargaville , A. Ohlsson , and R. Soll , “Surfactant for Meconium Aspiration Syndrome in Term and Late Preterm Infants,” Cochrane Database of Systematic Reviews 12 (2014): CD002054. 10.1002/14651858.CD002054.pub3 PMC7027383 25504256

