# Supplementary material for: Pulmonary Surfactant Treatment for Preterm Infants With Respiratory Distress Syndrome: A Nationwide Survey
Source: Clin Respir J. 2026 Sep 1;20(9):e70228. doi: 10.1111/crj.70228 (PMC13534843; doi:10.1111/crj.70228)
Supplement: Supplementary file 1 [file CRJ-20-e70228-s001.docx]

**Supplementary materials**

[Table S1 Characteristics of physicians and hospitals included in the survey	2](#_Toc185414851)

[Table S2. Availability of surfactants in different regions	3](#_Toc185414852)

[Table S3 FiO_2_ as a criterion for surfactant administration in patients under non-invasive ventilation in different hospitals	4](#_Toc185414853)

[Supplemental 1-MUNICH questionnaire	5](#_Toc185414854)

# Table S1 Characteristics of physicians and hospitals included in the survey

|  | **Population (N=394)** |
| --- | --- |
| **Sex, n (%)** |  |
| Male | 151 (38.3) |
| Female | 243 (61.7) |
| **Age (years)** |  |
| **Median (Q1, Q3)** | **43.0 (39.0, 50.0)** |
| **Hospital type, n (%)** |  |
| Maternity-child healthcare hospital | 137 (34.8) |
| Children's hospital | 16 (4.1) |
| General hospital | 241 (61.2) |
| **Hospital level, n (%)** |  |
| Tertiary hospital | 240 (60.9) |
| Secondary hospital | 154 (39.0) |
| **City tier, n (%)** |  |
| 1st | 131 (33.2) |
| 2^nd^ | 55 (14.0) |
| 3^rd^ and lower-tier | 208 (52.8) |
| **Geographical region** |  |
| Northeast China | 39 (9.9) |
| North China | 49 (12.4) |
| East China | 72 (18.3) |
| South China | 53 (13.5) |
| Central China | 55 (14.0) |
| Northwest China | 53 (13.5) |
| Southwest China | 73 (18.5) |
| **Doctor’ title** |  |
| Chief and associate chief physician | 273 (69.3) |
| Attending and resident physician | 121 (30.7) |
| **Department, n (%)** |  |
| Neonatology | 372 (94.4) |
| NICU | 12 (3.0) |
| Other | 10 (2.5) |

Abbreviations: NICU, neonatal intensive care unit.

# Table S2. Availability of surfactants in different regions

|  | **Total (n=394)** | **Geographical regions** | | | | | | | |
| --- | --- | --- | --- | --- | --- | --- | --- | --- | --- |
|  |  | **Northeast China** | **North China** | **East**  **China** | **South China** | **Central China** | **Northwest China** | **Southwest China** | ***p*** |
| **Poractant alfa,** n (%) | 349 (88.6) | 33 (84.6) | 40 (81.6) | 61 (84.7) | 51 (96.2) | 53 (96.4) | 46 (86.8) | 65 (89.0) | 0.104 |
| **Calsurf,** n(%) | 229 (58.1) | 27 (69.2) | 36 (73.5) | 39 (54.2) | 24 (45.3) | 37 (67.3) | 32 (60.4) | 34 (46.6) | 0.010 |
| **No surfactant,** n(%) | 13 (3.3) | 2 (5.1) | 2 (4.1) | 1 (1.4) | 1 (1.9) | 1 (1.8) | 2 (3.8) | 4 (5.5) | 0.788 |

# Table S3 FiO_2_ as a criterion for surfactant administration in patients under non-invasive ventilation in different hospitals

|  |  | **Hospital tier** | | | **Hospital type** | | | | **City tier** | | | |
| --- | --- | --- | --- | --- | --- | --- | --- | --- | --- | --- | --- | --- |
|  | **N=238** | **Tertiary hospital** | **Secondary hospital** | ***p*** | | **General hospital** | **Paediatric specialty hospital** | ***p*** | **1st-tier cities** | **2nd-tier cities** | **3rd-tier and lower-tier cities** | ***p*** |
| >21% | 4 (1.7) | 2 (1.1) | 2 (3.5) | 0.062 | | 4 (2.9) | 0 | 0.165 | 0 | 1 (3.4) | 3 (2.4) | 0.077 |
| >30% | 99 (41.6) | 83 (45.9) | 16 (28.1) |  | | 52 (37.4) | 47 (47.5) |  | 41 (48.8) | 9 (31.0) | 49 (39.2) |  |
| >40% | 98 (41.2) | 73 (40.3) | 25 (43.9) |  | | 57 (41.0) | 41 (41.4) |  | 34 (40.5) | 16 (55.2) | 48 (38.4) |  |
| >50% | 12 (5.0) | 7 (3.9) | 5 (8.8) |  | | 8 (5.8) | 4 (4.0) |  | 5 (6.0) | 2 (6.9) | 5 (4.0) |  |
| >60% | 25 (10.5) | 16 (8.8) | 9 (15.8) |  | | 18 (12.9) | 7 (7.1) |  | 4 (4.8) | 1 (3.4) | 20 (16.0) |  |

# Supplemental 1-MUNICH questionnaire

**Shortened Informed Consent**

**Dear Sir/Madam:**

We invite you to join a project called "*A National Cross-sectional Survey on the Status Quo of Management of Neonatal Respiratory Distress Syndrome* ". Please read the following carefully before you start filling in the questionnaire. This informed consent can help you learn about information related to the project, as well as your benefits and responsibilities.

**Project introduction:**

This survey is a cross-sectional study of approximately 450 clinicians engaged in the diagnosis and treatment of neonatal respiratory distress syndrome (RDS) in China. The purpose is to present the status quo of neonatal RDS diagnosis and treatment ideas and behavior in China, which can help optimize the clinical management of neonatal RDS in the future, so as to improve the prognosis of children with RDS.

**Information confidentiality:**

The information you submit through the questionnaire will be regarded as your personal information and stored in the database. All project-related personnel are required to keep your personal information confidential. If necessary, only government administration authorities can access your information.

**Participation in, refusal of, and withdrawal from the survey:**

You voluntarily choose to refuse or participate in the survey, and you can withdraw from the survey at any time during the process without any reason. Your decision will not have any impact on your diagnosis and treatment.

(If you want to learn more about the content of this project, please click on the link below.)

<https://docs.qq.com/doc/p/e585ab6ba5bbff1a9a70d59eb816a7f0996e41aa?dver=3.0.0>

**Informed consent statement:**

I have read the above introduction to this survey and am fully aware of the risks and benefits that may arise from participating in this survey. I voluntarily agree to participate in this survey.

I agree □ disagree □

Signature	Date

**Questionnaire on the Status Quo of Clinical Management of Neonatal Respiratory Distress Syndrome in China**

**(MUNICH Questionnaire)**

**Version No.: DV1.0**

**Version Date: 2022-09-10**

**Filling instructions:**

**Hello! Thank you very much for taking the time to participate in this survey. The purpose of this survey is to learn about the status quo of the management of neonatal respiratory distress syndrome (RDS) in China. This survey is anonymous. Please try your best to make the choice that best suits your situation. Any information you provide will be kept strictly confidential. Thank you for your support!**

**(Note: If you choose the option "Other" for a question, you should provide the corresponding answer on the horizontal line after "please specify". Otherwise, the questionnaire cannot be submitted.)**

Your region: (Fill-in-the-blank)

Province ____________

City ____________

The type of your hospital: (Single choice)

A. Maternity-child healthcare hospitals

B. Children's hospital

C. General hospital

D. Other, please specify ____________

Does your hospital have an obstetrics department? (Single choice)

A. Yes

B. No

The level of your hospital is: ________ (Single choice)

A. 3A

B. 3B

C. 2A

D. 2B

E. Other, please specify ____________

The number of beds in the neonatology department at your hospital is: _________ (Fill-in-the-blank)

**Formal Questionnaire**

1. **Basic Information**
1.1 Your gender: (Single choice)
A. Male
B. Female
1.2 Your age: ______ (Fill-in-the-blank)
1.3 The name of your hospital: _________ (Fill-in-the-blank)
1.4 Your professional title: (Single choice)

A. Chief physician

B. Associate chief physician

C. Attending physician

D. Resident physician

1.5 Your department/division: ________ (Fill-in-the-blank)
1.6 Your time of service in the specialty of neonatology: (Fill-in-the-blank)

______ year(s) and ______ month(s)

1.7 Neonatology at your hospital is: (Multiple-choice)

A. An independent department/division

B. A unified ward, with no separate neonatal intensive care unit (NICU)

C. Merged with general pediatrics

D. Divided into NICU and a general neonatal ward

E. Other, please specify ____________________

1.8 (a) The proportion of out-patient is: ______% (If there are no out-of-hospital patients, please fill in "0")

1.8	(b) The number of NICU beds at your hospital is: ____

1.8	(c) The average number of RDS patients admitted per week is: ____ (Fill-in-the-blank)

1.9 The number of doctors in neonatology department is: ___, and the number of nurses is: ___ (Fill-in-the-blank)

**2. Prenatal Care and Delivery Room Stabilization**

2.1 Percentage of prenatal steroids use (any dosage) in preterm infants < 34 weeks of gestational age (GA): _______%; inborn patients: _______%; outborn patients: _______%. (Fill-in-the-blank, if there are no neonates with RDS, please fill in "none")
2.2 What do you think are the reasons that prenatal steroids are not used in preterm infants < 34 weeks of GA? Please sort them in descending order of importance according to the actual clinical situation. (If none of the following reasons are common, supplementary explanations may be provided in the "Other" option.) (Sorting)

[	] Obstetricians don't pay enough attention despite awareness

[	] Precipitous labour with no time to use

[	] Accessibility of medicines

[	] Little awareness of obstetricians

[	] Other, please specify ________________

2.3 Do you have oxygen blender for neonatal resuscitation in the delivery room (DR) of your hospital? (Single choice)

A. Yes

B. No

2.3 (a) What is the initial setting of fraction of inspired oxygen (FiO_2_) for resuscitation? (Fill-in-the-blank)

Neonates born at <28 weeks of GA: _______; Neonates born at 28 to 32 weeks of GA: ________; Neonates born at >32 weeks of GA: ________

2.3 (b) The initial FiO_2_ for resuscitation is: _____ (Fill-in-the-blank)

2.4 What are available for positive pressure ventilation in the DR of your hospital? (Multiple-select)
A. T-piece resuscitator
B. Continuous positive airway pressure (CPAP)
C. Bilevel positive airway pressure (BiPAP)
D. Nasal intermittent positive pressure ventilation (NIPPV)
E. Heated humidified high-flow nasal cannula (HHHFNC)
F. Bag-valve-mask resuscitators
G. Others, please specify ____
H. Positive pressure ventilation is currently unavailable

2.4 (a) What are the reasons that only bag-valve-mask resuscitators can be used in the DR? (Multiple-select)

A. Lack of equipment

B. No suitable air-oxygen interface

C. Personnel and technical limitations

D. Other, please specify ____________________

2.5 What is the timing of endotracheal intubation during resuscitation in preterm infants? (Multiple-select)

A. Extremely preterm infants who have severe dyspnea after birth

B. Extremely low birth weight preterm infants

C. Preterm infants <____ weeks of GA

D. The target heart rate or SpO_2_ cannot be reached after T-piece or bag-valve-mask resuscitation

E. No spontaneous breathing or poor spontaneous breathing is detected

F. Tracheal administration (of pulmonary surfactants (PS)/epinephrine) is required

G. Other, please specify ____________________

2.6 What measures do you take to prevent hypothermia in the DR and during transportation between the DR and NICU? (Multiple-select)

A. Room temperature adjustment in the DR

B. Swaddling

C. Radiant warmer heating

D. Plastic wrapping

E. Use of an electric blanket

F. Transport incubator preheating

G. Temperature adjustment in transportation areas

H. Preheating of equipment (such as ultrasonic couplants and disinfectants)

I. Other, please specify ____________________

2.7 I**s** pulmonary surfactant (PS) available in the DR of your hospital? (Single choice)

A. Yes

B. No

2.7 (a) Under what circumstances will you administrate PS in DR? (Multiple-select and fill-in-the-blank)

A. Preterm infants born <___ weeks of GA

B. When endotracheal intubation is required for resuscitation

C. FiO_2_ > 30% under non-invasive ventilation

D. Other, please specify ____________________

2.7 (b) What are the reasons that PS is not available in the DR? Please sort them in descending order of importance according to the actual clinical situation. (If none of the following reasons are common, supplementary explanations may be provided in the "Other" option.) (Sorting)

A. Parents lack willingness to save the preterm babies

B. PS need to be refrigerated and cannot be stored in the DR

C. Medicines purchased before the birth of an infant are not covered by medical insurance

D. Pharmacies are unable to lend medicines in advance

E. Parents may refuse to pay after borrowing medicines from pharmacy

F. Collaboration between physicians and nurses for PS administration in the DR is poor

G. Other, please specify ____________________

2.8 For women at risk of preterm labour, if prenatal consultations are available? (Single choice)

A. Yes

B. No

2.8 (a) Prenatal consultations are offered for those <__ weeks who are at risk of preterm labour. (Fill-in-the-blank)

2.9 In your opinion, the target oxygenation of preterm infants after resuscitation should be ____% to ____%. (Fill-in-the-blank)
2.10 For extremely preterm infants born < 28 weeks of GA, do you think the resuscitation in the DR are roughly the same for different gestational ages? (Single choice)

A. Yes

B. No

2.10 (a) In your opinion, treatment can be considered in those ≥ ____ weeks of GA and active

treatment will be given in those ≥ ____ weeks. (Fill-in-the-blank)

2.10 (b) During resuscitation, endotracheal intubation will be performed directly after birth in

patients < ____ weeks of GA. (Fill-in-the-blank)

2.11 Does your hospital routinely perform group B *Streptococcus* screening (GBS)? (Single choice)

A. Yes

B. No

2.11 (a) Will GBS screening be done for pregnant women who have not yet completed GBS

screening but are at risk of preterm labour? (Single choice)

A. Yes

B. No

**3. Non-invasive Ventilation**

3.1 What modes of non-invasive ventilation (NIV) are available at your hospital? (Multiple-choice)

A. Heated humidified high-flow nasal cannula (HHHFNC)

B. Continuous positive airway pressure (CPAP)

C. Bilevel positive airway pressure (BiPAP)

D. Nasal intermittent positive pressure ventilation (NIPPV)

E. Non-invasive high-frequency oscillation (nHFO)

3.2 The proportion of RDS patients who have non-invasive respiratory support as their primary respiratory support is approximately: _____%. (Fill-in-the-blank)
3.3 Which mode is your first choice for primary non-invasive respiratory support? Please sort them in descending order of use frequency according to the actual clinical situation. (If none of the following modes are used, supplementary explanations may be provided in the "Other" option.) (Sorting)

[	] Heated humidified high-flow nasal cannula (HHHFNC)

[	] Continuous positive airway pressure (CPAP)

[	] Bilevel positive airway pressure (BiPAP)

[	] Nasal intermittent positive pressure ventilation (NIPPV)

[	] Non-invasive high-frequency oscillation (nHFO)

[	] Other, please specify ____________________

3.4 (a) What do you think are the advantages of nasal intermittent positive pressure ventilation (NIPPV) over CPAP? (Multiple-choice)

A. Improve ventilation

B. Reduce intubation

C. Reduce bronchopulmonary dysplasia (BPD) in preterm infants

D. Reduce NIV failures

E. Other, please specify ____________________

3.4 (b) What do you think are the advantages of non-invasive high-frequency oscillation (nHFO) over CPAP? (Multiple-choice)

A. Improve ventilation

B. Reduce intubation

C. Reduce bronchopulmonary dysplasia (BPD) in preterm infants

D. Reduce NIV failures

E. Other, please specify ____________________

3.5 What do you think are the disadvantages of NIPPV and nHFO? (Multiple-choice)

A. Thick secretions

B. Air leaks

C. Abdominal distension

D. Nasal injuries

E. Necrotizing enterocolitis (NEC)

F. Ventilator-associated pneumonia

G. Intraventricular hemorrhage (IVH)

H. Other, please specify ____

3.6 For RDS patients, initial positive end-expiratory pressure (PEEP) of continuous positive airway pressure (CPAP) would usually be ___ to ___ cmH_2_O? (Fill-in-the-blank)
3.7 Which mode do you prefer for preterm babies weaning from ventilators? Please sort them in descending order of use frequency according to the actual clinical situation. (If none of the following modes are used, supplementary explanations may be provided in the "Other" option.) (Sorting)

[	] Heated humidified high-flow nasal cannula (HHHFNC)

[	] Continuous positive airway pressure (CPAP)

[	] Bilevel positive airway pressure (BiPAP)

[	] Nasal intermittent positive pressure ventilation (NIPPV)

[	] Non-invasive high-frequency oscillation (nHFO)

[	] Other, please specify ____________________

**4. Mechanical Ventilation**

4.1 Under what circumstances do you think RDS patients need mechanical ventilation? (Multiple-choice)

A. Severe symptoms

B. Blood gas analysis suggests hypercapnia

C. High positive pressure and/or FiO_2_ are/is required for maintaining target oxygenation

D. X-ray suggests grade 3 to 4 RDS

E. Oxygenation and heart rate cannot be maintained

F. Frequent apnea

G. Other, please specify ____________________

4.2 Which mode of mechanical ventilation is your preference? Please sort them in descending order of use frequency according to the actual clinical situation. (If none of the following modes are used, supplementary explanations may be provided in the "Other" option.) (Sorting)

[	] Pressure control (PC-AC) + Volume guarantee (VG)

[	] Pressure control (PC-AC)

[	] Synchronized intermittent mandatory ventilation (SIMV) + Pressure support (PSV) + Volume guarantee (VG)

[	] Synchronized intermittent mandatory ventilation (SIMV) + Pressure support (PSV)

[	] High-frequency oscillatory ventilation (HFOV) + Volume guarantee (VG)

[	] High-frequency oscillatory ventilation (HFOV)

[	] Other, please specify ____________________

4.2 (a) Under what circumstances will high-frequency oscillatory ventilation be preferred? (Multiple-choice)

A. Very preterm infants requiring invasive ventilation

B. Extremely preterm infants requiring invasive ventilation

C. Very low birth weight infants requiring invasive ventilation

D. Extremely low birth weight infants requiring invasive ventilation

E. Rescue therapy after failure of conventional mechanical ventilation

F. Pulmonary hemorrhage

G. Meconium aspiration syndrome (MAS)

H. Diaphragmatic hernia

I. Persistent pulmonary hypertension of the newborn (PPHN)

J. Other, please specify ________________________

4.3 Do you use caffeine during mechanical ventilation? (Multiple-choice)
A. Routine
B. Before weaning from ventilation
C. No use
4.4 Do you think some preterm infants should start caffeine therapy **as early as** possible after birth? (Single choice)

A. Yes

B. No

4.4 (a) You will start caffeine therapy for preterm infants ≤ ____ weeks of GA or with birth weight ≤ _____ g as early as possible after birth. Usually, a _____ mg/kg loading dose and a _____ to _____ mg/kg maintenance dose of caffeine citrate injection are administered. (Fill-in-the-blank)

4.5 When do you think caffeine should be stopped? (Multiple-choice)

A. No apnea for 5 to 7 days

B. Corrected gestational age 33 to 35 weeks

C. Corrected gestational age 33 to 35 weeks and no apnea for 5 to 7 days

D. Corrected gestational age 33 to 35 weeks, weaned from positive pressure ventilation, and no apnea for 5 to 7 days

E. Used until 37 weeks and above if respiratory support is required

F. Other, please specify ____________________

4.6 What is your corticosteroids therapy for management of bronchopulmonary dysplasia (BPD) in preterm infants? (Multiple-choice)

| A. 8 to 14 days, < 2 mg/kg (cumulative dose), intravenous dexamethasone injection | B. 8 to 14 days, 2 to 4 mg/kg (cumulative dose), intravenous dexamethasone injection |
| --- | --- |
| C. 8 to 14 days, > 4 mg/kg (cumulative dose), intravenous dexamethasone injection | D. 15 to 27 days, < 2 mg/kg (cumulative dose), intravenous dexamethasone injection |
| E. 15 to 27 days, 2 to 4 mg/kg (cumulative dose), intravenous dexamethasone injection | F. 15 to 27 days, > 4 mg/kg (cumulative dose), intravenous dexamethasone injection |
| G. < 8 days, intravenous hydrocortisone injection | H. ≥ 8 days, intravenous hydrocortisone injection |
| I. < 8 days, inhaled budesonide suspension | J. ≥ 8 days, inhaled budesonide suspension |
| K. Intratracheal budesonide suspension (combined with PS) | L. 8 days, inhaled fluticasone suspension |
| M. < 8 days, inhaled beclomethasone suspension | N. ≥ 8 days, inhaled beclomethasone suspension |
| O. Other, please specify _____ |  |

**5. PS Administration**

5.1 What are the **prerequisites** for your diagnosis of neonatal RDS? (Multiple-choice)

A. Medical history

B. Clinical symptoms

C. X-ray

D. Imaging such as lung ultrasound

E. Blood gas analysis

F. Other, please specify ____________________

5.2 Will you use FiO_2_ as the predictior for PS administration in RDS patients under non-invasive ventilation? (Single choice)

A. Yes

B. No

5.2 (a) FiO_2_ threshold for using PS? (Single choice)

A. ＞ 21%

B. ＞ 30%

C. ＞ 40%

D. ＞ 50%

E. ＞ 60%

F. Other, please specify ____________________

5.3 **Do** you routinely use PS for RDS patients who need endotracheal intubation after NIV failure? (Single choice)

A. Yes

B. No

5.4 Available PS at your hospital? (Multiple-choice)

A. Porcine PS

B. Bovine PS

C. Other, please specify ____________________

5.4 (a) Initial dose of porcine PS? (Single choice)

A. 200 mg/kg

B. 100 to 200 mg/kg

C. 100 mg/kg

D. < 100 mg/kg

E. > 200 mg/kg

5.4 (b) Initial dose of bovine PS? (Single choice)

A. 100 mg/kg

B. 70 mg/kg

C. 40 mg/kg

D. 40 to 100 mg/kg

E. > 100 mg/kg

F. < 40 mg/kg

5.5 What are the main factors considering for choosing different PS products? Please select 5 main factors according to the actual clinical situation, and sort them in descending order of importance. (If none of the following factors are considered, supplementary explanations may be provided in the "Other" option.) (Sorting)

[	] Gestational age

[	] Efficacy

[	] Severity of illness

[	] Method of administration

[	] Time of administration

[	] Access to medicines

[	] Price

[	] Countries of origin

[	] Source of derivation (porcine/bovine)

[	] Medical insurance

[	] Other, please specify ____________________

5.6 (a) You will choose porcine PSfor preterm infants ≤ ___ weeks of GA? (Fill-in-the-blank)

(b) The proportion of surfactant use in moderate preterm infants (32 weeks ≤ GA < 33^+6^ weeks) with RDS is approximately ___%. (Fill-in-the-blank)

(c) The proportion of surfactant use in late preterm infants (34 weeks ≤ GA < 36^+6^ weeks) with RDS is approximately ___%. (Fill-in-the-blank)

5.7 Methods of PS administration for RDS patients under non-invasive ventilation? (If none of the following methods are used, supplementary explanations may be provided in the "Other" option.) (Multiple-choice)

A. Intubation–surfactant–extubation (InSurE)

B. Less invasive surfactant administration/Minimally invasive surfactant therapy (LISA/MIST)

C. Invasive mechanical ventilation after endotracheal administration

D. Other, please specify ____________________

5.8 (a) For INSURE method, endotracheal intubation can be removed _____ minutes after PS administration? (Fill-in-the-blank)

(b) If LISA/MIST is used, the actual proportion is roughly ____% of RDS patients under NIV. (Fill-in-the-blank)

(c) When using LISA/MIST for PS administration, your choice of tubes? (Multiple-choice)

A. Gastric tube

B. Umbilical arterial/venous catheter

C. Peripheral vein catheters

D. Other, please specify ____________________

5.9 Do you think **a higher initial PS dose** (package insert recommended) is more effective? (Single choice)

A. Yes

B. No

5.10 Under what circumstances would you consider using **a higher dose of PS** (package insert recommended)? (Multiple-choice)

A. Smaller gestational age

B. Severe clinical manifestations

C. Grade 3 to 4 RDS in X-ray

D. No prenatal steroid

E. No tendency

F. A higher PS dose is selected in any situation

G. Other, please specify ____________________

5.11 In your opinion, the first PS dose is generally administered within ____ hours after birth, the time from the extraction of porcine PS to the completion of injection is: ____ minutes, and the time from the vibration and dissolution of bovine PS to the completion of injection is: ____ minutes. (Fill-in-the-blank)
5.12 Will you administer **PS in combination with glucocorticoids**? (Single choice)

A. Yes

B. No

5.12 (a) When do you think **PS combined with glucocorticoids** should be administered? (Multiple-choice)

A. **First** **dose** of surfactant

B. **Second** **dose** of surfactant

C. Other, please specify ____________________

5.13 When will you consider to administer **the second dose of PS**? (Multiple-choice)

A. No improvement in dyspnea

B. Ventilator parameters (mean airway pressure and FiO_2_) are high

C. No significant improvement in oxygenation

D. Chest radiography shows no significant improvement

E. Blood gas analysis still suggests hypercapnia

F. Other, please specify ____________________

5.14 In your opinion, if **a second dose of PS** is needed, the interval time should be _____ hours from the first dose. (Fill-in-the-blank)
5.15 PS can be used in diseases including_____? (Multiple-choice)

A. RDS infants following a cesarean section, especially an elective cesarean section

B. RDS infants whose mothers have diabetes

C. Acute lung injury (ALI) due to severe neonatal asphyxia

D. Acute lung injury (ALI) due to severe infectious pneumonia

E. Acute lung injury (ALI) due to severe meconium aspiration syndrome

F. Acute lung injury (ALI) caused by ALI due to pulmonary hemorrhage, etc.

G. RDS infants caused by a genetic defect in the SP-B or SP-C gene

H. Acute respiratory distress syndrome (ARDS) in newborn

I. Other, please specify ____________________

**6. Others**

6.1 What are the treatments available for RDS infants complicated with persistent pulmonary hypertension of the newborn (PPHN) at your hospital? (Multiple-choice)

| A. Nitric oxide | B. Sildenafil |
| --- | --- |
| C. Bosentan | D. Inhaled prostacyclin |
| E. Milrinone | F. Mechanical ventilation |
| G. Vasoactive drugs | H. Transfusion of blood products |
| I. Volume expansion with a saline solution | J. Use of PS |

6.2 Is **bedside lung ultrasound** available in your hospital? (Single choice)

A. Yes

B. No

6.2 (a) What are the application scenarios of **lung ultrasound** in RDS management at your hospital? (Multiple-choice)

A. RDS diagnosis and identification

B. PS administration prediction and efficacy monitoring

C. BPD prediction

D. Diagnosis of complications

E. Other, please specify ____________________

6.2 (b) What are the reasons that **lung ultrasound** is not used at your hospital? (Multiple-choice)

A. No suitable ultrasound machine

B. No relevant technical personnel in the department

C. No corresponding examination item in the department of ultrasound

D. Inability to issue a formal report

E. Inability to collect payment

F. Other, please specify ____________________

6.3 Does your hospital have a breast milk bank where breast milk can be tested, screened, sterilized, safely stored, and distributed? (Single choice)

A. Yes

B. No

6.4 Is there a home-like ward at your hospital? (Single choice)

A. Yes

B. No

6.4 (a) The number of beds in your home-like ward is: ____ (Fill-in-the-blank)

6.5 Does your hospital offer family integrated care? (Single choice)

A. Yes

B. No

6.6 What do you think are the common reasons that parents decide not to continue treatment in NICU for extremely preterm infants < 28 weeks of GA? Please sort them in descending order of importance. (If none of the following reasons are common, supplementary explanations may be provided in the "Other" option.) (Sorting)

[	] Financial reasons

[	] Sequelae of brain injury

[	] Family stress

[	] Illness severeness

[	] Social opinion factors

[	] Other, please specify ____

6.7 Do you know that **PS are listed in category B** of the national reimbursement drug list? (Single choice)

A. Yes

B. No

6.8 The proportion of RDS patients who have medical insurance at your hospital is approximately: ____%, and the proportion of medical insurance reimbursements to hospitalization costs is approximately: ____%. (Fill-in-the-blank)
6.9 What guidelines/consensuses do you follow in the treatment of RDS? (Multiple- choice)

A. Subspecialty Group of Neonatology, the Society of Pediatrics, Chinese Medical Association:

Consensus for Pulmonary Surfactant Therapy in Neonates in China

B. European Society for Paediatric Research: European Consensus Guidelines on the Management of Neonatal Respiratory Distress Syndrome in Preterm Infants – 2019 Update

C. American Academy of Pediatrics: Respiratory Support in Preterm Infants at Birth

D. Canadian Paediatric Society: Guidelines for Surfactant Replacement Therapy in Neonates

E. National Institute for Health and Care Excellence: Specialist Neonatal Respiratory Care for Babies Born Preterm

F. Other, please specify ________________

6.10 What difficulties have you encountered in the clinical application of the guidelines? (Multiple-choice)

A. Facilities in the DR are inadequate

B. Respiratory support equipment is backward

C. The number of respiratory support equipment is insufficient

D. Medicines are expensive and difficult to access

E. Ideas are outdated and hard to change

F. The financial burden is too heavy for parents to bear

G. Examinations are difficult to obtain, and the waiting time is long

H. Other, please specify ________________

6.11 What are your comments and suggestions for better clinical practices? Please sort them in descending order of importance. (Sorting)

[	] Surfactant storage, use, and reimbursement in the DR

[	] Positive pressure ventilation equipment, transport equipment such as integrated transport incubator, etc., in the DR

[	] Less invasive surfactant administration/Minimally invasive surfactant therapy (LISA/MIST) training

[	] Strengthening of multidisciplinary collaboration between neonatal and obstetric specialties, and standardization of the resuscitation

[	] Promotion of lung ultrasound

[	] Application of volume guaranteed mode

[	] Other, please specify ________________

6.12 What RDS-related research are your interests? Please sort them in descending order of importance. (Sorting)

[	] Lung ultrasound

[	] BPD prevention and treatment

[	] Umbilical cord blood stem cells

[	] Surfactant administration in combination with steroids

[	] Less invasive surfactant administration/Minimally invasive surfactant therapy (LISA/MIST)

[	] Non-invasive high-frequency oscillation

[	] Other, please specify ________________
